# Synthesis and characterization of Anderson-Evans type polyoxometalates, antibacterial properties

**DOI:** 10.55730/1300-0527.3575

**Published:** 2023-06-07

**Authors:** Hülya AVCI ÖZBEK

**Affiliations:** Department of Chemistry, Faculty of Sciences and Liberal Arts, Manisa Celal Bayar University, Manisa, Turkiye

**Keywords:** Aluminum-substituted, Anderson-Evans polyoxometalate, antimicrobial activity

## Abstract

In the present work, the new aluminium-substituted polyoxometalates of the Anderson-Evans type have been prepared and structurally defined by the reaction of aluminium (III) chloride hexahydrate and sodium tungstate dihydrate/sodium molybdate dihydrate in an aqueous basic medium. Elemental analysis, FT-IR, TGA, ^1^H NMR, and ^31^P NMR analysis revealed that these polyoxometalates had the following formula: [Ph_4_P]_3_[Al(OH)_6_Mo_6_O_18_]·4H_2_O **1**, [Ph_4_P]_3_[Al(OH)_6_W_6_O_18_]·4H_2_O **2**, [C_7_H_10_N]_3_[Al(OH)_6_Mo_6_O_18_]·4H_2_O **3**, [C_7_H_10_N]_3_[Al(OH)_6_W_6_O_18_]·4H_2_O **4**. The compounds **1** and **2** show promising antibacterial activity against gram-positive *Staphylcoccus aureus* ATCC 25923 and gram-negative *Escherichia coli* ATCC 25922 bacteria.

## 1. Introduction

Polyoxometalates (POMs) are metal oxide polyanionic clusters. A large number of POMs have been reported since the first POM, (NH_4_)_3_[PMo_12_O_40_], was reported by Berzelius in 1826. These numerous POMs compositions and structural architectures are generally divided into two fundamental classes such as isopolyanions [M_x_O_y_]^m−^ and heteropolyanions [X_z_M_x_O_y_]^n−^. Among these heteropolyanions, the Keggin, Anderson-Evans and Wells-Dawson types are the most widely studied in the literature [1–7]. The chemistry of POMs is an extensive and growing area of modern coordination chemistry. Much attention has been focused on engineering materials, photocatalysis, nanotechnology, catalysis, electrochemistry, biochemistry, and medicine [8–31].

Anderson-Evans type POMs are an important subfamily among the polyoxometalates because they are composed of a single metal atom supported by a polytungstate or polymolybdate. There are two types of Anderson-Evans POM: The general formula of the A-type is [X^n+^M_6_O_24_]^(12-n)−^ with the heteroatom in its highest oxidation state (e.g., Sb^5+^, Te^6+^, I^7+^), the B-type has the general formula [X^n+^(OH)_6_M_6_O_18_]^(6-n)−^ where the heteroatom has lower oxidation states (e.g., Co^2+^, Ni^2+^, Al^3+^, Fe^3+^) [5]. In the B-type hydroxy groups can be replaced by the organic ligands to obtain a new polyoxometalate, thus providing different modifications [32–35]. The binding tendency of metal ions is utilized to obtain Anderson-Evans type heteropolyanions. Due to the high binding tendency of aluminium metal, [Al(OH)_6_Mo_6_O_18_]^3−^ is used to obtain new materials. However, although there are many Anderson-Evans type POMs, studies on aluminium-containing Anderson-Evans type structures are very limited in the literature. In this paper, it is aimed to provide a new perspective to the studies in the literature with the synthesis and antimicrobial applications of new aluminium-containing Anderson-Evans type compounds. When we examine the studies on this subject in the literature, we come across the following studies: [Ln(H_2_O)_7_{Al(OH)_6_Mo_6_O_18_}]·yH_2_O [36], (C_6_H_10_N_3_O_2_)_2_Na(H_2_O)_2_[Al(OH)_6_Mo_6_O_18_]·6H_2_O [37], [Al(H_2_O)_6_][Al(OH)_6_Mo_6_O_18_]·10H_2_O [38], Na_0.5_Cs_1.75_[H_0.25_Fe_0.25_Al(OH)_6_Mo_6_O_18_]·8H_2_O [39], [Al(OH)_6_Mo_6_O_18_{Cu(Phen)(H_2_O)_2_}_2_][Al(OH)_6_Mo_6_O_18_{Cu(Phen)(H_2_O)Cl}_2_]·5H_2_O [40], [Cu^II^(2,2’−bipy)(H_2_O)_2_Al(OH)_6_Mo_6_O_18_]*_n_^n^*^−^ [41], [Eu(H_2_O)_7_][Al(OH)_6_Mo_6_O_18_]·4H_2_O and {(C_2_H_5_NO_2_)_2_[Eu(H_2_O)_5_]}[Al(OH)_6_Mo_6_O_18_]·10H_2_O [42], Na_2_(H_2_O)_4_(H_3_O)[Al(OH)_6_Mo_6_O_18_] [43].

In this study, four aluminum substituted Anderson-Evans type POMs were synthesized and fully characterized by spectroscopic methods. The antibacterial activity of these compounds was investigated.

## 2. Experimental section

### 2.1. General methods

All chemicals used in this study were all of analytical grade purchased from Sigma-Aldrich, or Merck and used without purification. ^1^H and ^31^P NMR spectra were recorded on a AVANCE III 400 MHz NaNoBay FT-NMR spectrometer operating at 400 MHz (^1^H), (^31^P) in DMSO-d_6_. FT-IR spectrum was obtained from a sample powder palletized with KBr on Perkin Elmer LR 64912 C spectrometer over the range 400–4000 cm^−1^. C, H and N elemental analyses were performed on a LECO-932 CHNS element analyzer. Thermogravimetric analysis (TGA) was carried out on a Hitachi Exstar TG/DTA 7300 thermal analyzer in flowing N_2_ between 25 and 800 °C at a heating rate of 10 °C/min.

### 2.2. Synthesis of compounds

Two solutions were prepared separately. Solution 1: Na_2_MO_4_·2H_2_O (1.30 mmol) (M=Mo, W) was dissolved in water (10 mL) under stirring. Solution 2: AlCl_3_·6H_2_O (0.52 mmol) was dissolved in water (10 mL) under stirring. Solution 2 was added to solution 1. The resulting mixture was kept at 60 °C and acidified with glacial acetic acid. In 1–2 h at this temperature, the mixture was cooled to room temperature. After o-toluidine hydrochloride (excess) was dissolved in 2 mL water; Ph_4_PBr (excess) was dissolved in chloroform and precipitate was formed by dropwise addition. The solid was filtered, washed with H_2_O and finally dried under a vacuum at 60 ºC.

#### 2.2.1. [Ph_4_P]_3_[Al(OH)_6_Mo_6_O_18_]·4H_2_O (1)

Yield: 380 mg, 35%. FT-IR (KBr pellets): ʋ = 439 (s), 527 (s), 572 (m), 614 (m), 688 (m), 723 (s), 755 (m), 798 (m), 895 (m), 918 (m), 948 (s), 996 (m), 1111 (s), 1163 (m), 1187 (m), 1438 (s), 1483 (m), 1586 (m), 3172 (w) cm^−1^. Elem. Anal. Calcd. C_72_H_74_P_3_AlMo_6_O_28_ (2082,88 g/mol): C, 40.70, H, 3.58. found: C, 41.52, H, 5.58. TGA (loss of 4 H_2_O): calcd. 3.46%, found 3.28%, (loss of 3 PPh_4_): calcd. 48.88%, found 47.95%. ^1^H NMR (DMSO-d_6_): δ 7.72–7.99 (m, 60H, Ar). ^31^P NMR (DMSO-d_6_): δ 22.51.

#### 2.2.2. [Ph_4_P]_3_[Al(OH)_6_W_6_O_18_]·4H_2_O (2)

Yield: 278 mg, 20%. FT-IR (KBr pellets): ʋ = 442 (s), 572 (s), 647 (s), 756 (s), 892 (m), 922 (m), 943 (m), 1129 (m), 1310 (m), 1493(s), 1524 (m), 1591 (m), 1940 (w), 2569 (m), 3204 (w) cm^−1^. Elem. Anal. Calcd. C_72_H_74_P_3_AlW_6_O_28_ (2610,28 g/mol): C, 33.13, H, 2.86. found: C, 33.73, H, 2.56. TGA (loss of 4 H_2_O): calcd. 2.76%, found 2.47%, (loss of 3 PPh_4_): calcd. 39.00%, found 39.01%. ^1^H NMR (DMSO-d_6_): δ 7.73–8.01 (m, 60H, Ar). ^31^P NMR (DMSO-d_6_): δ 22.52.

#### 2.2.3. [C_7_H_10_N]_3_[Al(OH)_6_Mo_6_O_18_]·4H_2_O (3)

Yield: 260 mg, 38%. FT-IR (KBr pellets): ʋ = 432 (m), 526 (s), 688 (s), 721 (s), 800 (s), 889 (s), 915 (s), 996 (s), 1107 (s), 1188 (m), 1317 (m), 1435 (s), 1483 (m), 1585 (s), 3061 (m), 3445 (w) cm^−1^. Elem. Anal. Calcd. C_21_H_44_N_3_AlMo_6_O_28_ (1389.20 g/mol): C, 18.16, H, 3.19, N, 3.02. found: C, 18.27, H, 3.41, N, 3.00. TGA (loss of 4 H_2_O): calcd. 5.18%, found 5.56%, (loss of 3 C_7_H_10_N): calcd. 23.35%, found 23.25%. ^1^H NMR (DMSO-d_6_): δ 2.05 (s, 9H, Me), 3.34 (s, 6H, NH_2_), 6.49 (t, 6H, CH) 6.62 (d, 3H, CH), 6.90 (dd, 6H, CH and H^+^).

#### 2.2.4. [C_7_H_10_N]_3_[Al(OH)_6_W_6_O_18_]·4H_2_O (4)

Yield: 100 mg, 10%. FT-IR (KBr pellets): ʋ = 437 (m), 522 (m), 802 (s), 893 (m), 947 (m), 1110 (m), 1153 (m), 1224 (m), 1299 (m), 1493 (m), 1592 (m), 2580 (m), 2921 (m), 3204 (w) cm^−1^. Elem. Anal. Calcd. C_21_H_44_N_3_AlW_6_O_28_ (1916.60 g/mol): C, 13.16, H, 2.31, N, 2.19. found: C, 13.48, H, 2.83, N, 2.04. TGA (loss of 4 H_2_O): calcd. 3.76%, found 3.77%, (loss of 3 C_7_H_10_N): calcd. 23.37%, found 23.28%. ^1^H NMR (DMSO-d_6_): δ 2.20 (s, 9H, Me), 3.34 (s, 6H, NH_2_), 6.89 (s, 6H, CH), 6.98 (s, 3H, CH) 7.10 (dd, 6H, CH and H^+^).

### 2.3. Antibacterial tests

Antibacterial tests of all compounds (**1–4**) were carried out by disk diffusion method with gram-positive and gram-negative bacteria. *Staphylcoccus aureus* ATCC 25923 was used as gram-positive bacteria, whereas *Escherichia coli* ATCC 25922 was used as gram-negative bacteria. Bacteria stored at −20 °C in media containing 16% glycerol were inoculated on Mueller Hinton Agar (MHA) by streaking method and incubated at 37 °C for 16–24 h. After incubation, bacterial colonies of which purity was checked were selected and inoculated into sterile Mueller Hinton Broth media using sterile loops. Density was adjusted as Mc Farland 0.5 using DEN-1B Densitometer device. Sterile Mueller Hinton Broth medium was used as a control in the Mc Farland measurement. (McFarland 0.5: corresponds to 1–2.10^8^ bacteria.) Bacteria were inoculated into different petri dishes for each bacterium, covering the entire surface with sterile swab sticks. Fifteen minutes were waited. After 20 microliter samples were impregnated on the blank discs and the blank discs were placed in the petri dish, they were incubated for 16–24 h at 37 °C after waiting for another 15 min. (Sterile empty discs treated with DMSO served as a negative control whereas discs impregnated Erythromycin was used as a positive control.) After incubation, if there is a zone formed by the sample, it was observed and measured. Zone formation of the antibiotic used as a positive control was confirmed. Experiments were repeated three times.

## 3. Results and discussion

### 3.1. Synthesis and characterization

**Figure 1:**
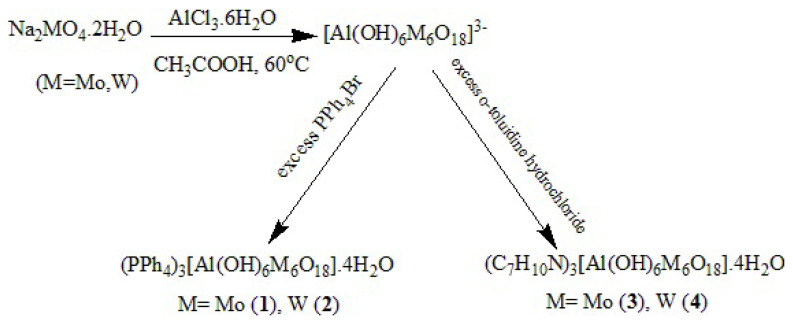
Synthesis of **1–4**.

Compounds **1–4** were synthesized by reaction of aluminum (III) chloride hexahydrate, sodium tungstate dihydrate/sodium molybdate dihydrate at temperature 60 °C in an acidic aqueous medium (Figure 1). The compounds were isolated as organo-soluble salts using counter ion (o-toluidinium chloride, tetraphenyl phosphonium bromide). The compounds were characterized by elemental analysis, ^1^H NMR, ^31^P NMR (for **1** and **2**), TGA and FT-IR.

#### 3.1.1. FT-IR

The FT-IR spectra (Figures S1–S4.) confirmed that these compounds (**1–4**) were an Anderson-type polyoxometalate, which had the same structure as the reported compounds [36–40, 44]. Two areas of characteristic peaks at 895–615 and 600–430 cm^−1^ are attributed to antisymmetric and symmetric deformation vibrations of M–O–M and M–O–Al (M=Mo, W) bridging fragments. The typical absorption peak of Anderson-Evans polyoxoanion is observed at about 948 (**1**), 943(**2**), 951(**3**), 947 (**4**) cm^−1^, which is attributed to the stretching vibration of terminal M=O units. For compounds **1** and **2**, the stretching vibrations between 995 and 1591 cm^−1^ were attributed to the Ph_4_P^+^; for compounds **3** and **4**, the stretching vibrations between 1107 and 1592 cm^−1^ were attributed to the C_7_H_10_N^+^. The broad bands at 3172 (**1**), 3204 (**2**), 3445 (**3**), 3204 (**4**) cm^−1^ of compounds (**1–4**) could be attributed to O-H bonds of crystalline water molecules.

#### 3.1.2. ^1^H and ^31^P NMR

The ^1^H NMR data for the compounds **1–4** in dimethyl sulfoxide (DMSO-d_6_) are presented in Figures S5–S8. The ^1^H NMR spectrum of **1** and **2** revealed phenyl protons (δ 7.72–7.99 **1**) (δ 7.73**–**8.01 **2**). **3** and **4** revealed the methyl protons (δ 2.05 **3**) (2.20 **4**); -NH_2_ signals (δ 3.34); methylene signals (δ 6.49, 6.62 and 6.90 **3**) (δ 6.89, 6.98 and 7.10 **4**). The ^1^H NMR results are in agreement with those from previous study [9,45]. The ^31^P NMR spectrum of **1** and **2** were recorded in dimethyl sulfoxide (DMSO-d_6_) and are depicted in Figures S9 and S10. The sharp and singlet peak appearing in the ^31^P NMR spectra, both with **1** and **2** cation resonances at 22.51 and 22.52 ppm respectively, can be attributed to Ph_4_P^+^ [9,45].

#### 3.1.3. TGA

In order to characterize the thermal stability of compounds **1–4**, their thermal behavior was investigated by TGA. In this investigation, heating rates were suitably controlled at 10 °C min^−1^ under N_2_ atmosphere and the weight loss was measured from ambient temperature up to 800 °C. The thermal behavior of all metal compounds is generally similar. Firstly, the thermograms of **1–4** (Figures S11–S14) show weight loss which starts at room temperature with a dehydration corresponding to the loss of four crystal water molecules. Table 1 shows the other degradation products for compounds **1–4** and the TGA results obtained are in agreement with the calculated value.

### 3.2. Antibacterial analysis

**Figure 2:**
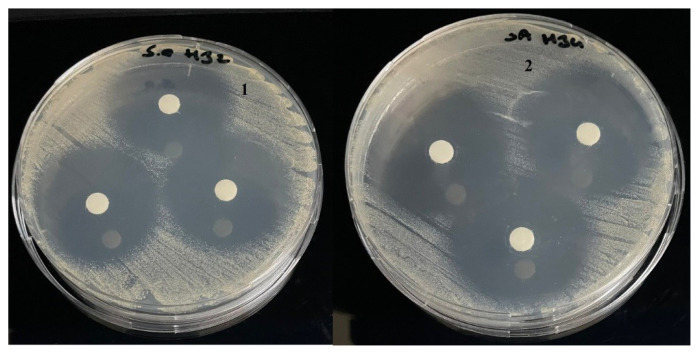
Antibacterial activity of **1–2** against *Staphylcoccus aureus* ATCC 25923.

**Figure 3:**
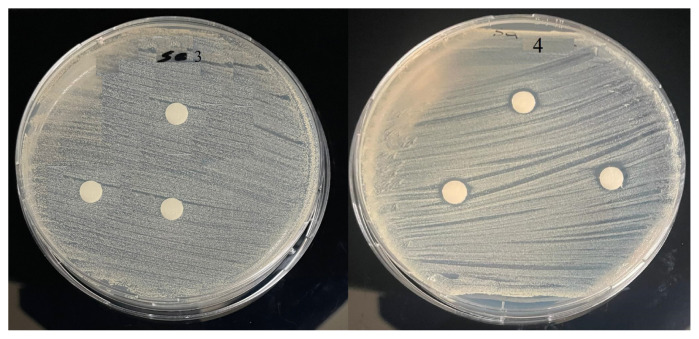
Antibacterial activity of **3–4** against *Staphylcoccus aureus* ATCC 25923.

**Figure 4:**
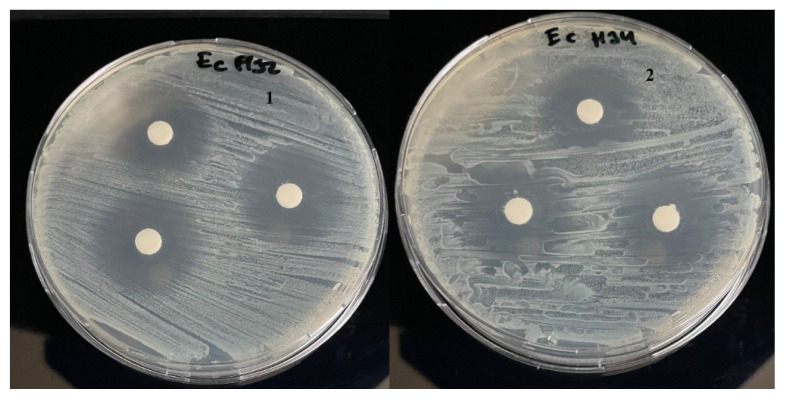
Antibacterial activity of **1–2** against *Escherichia coli* ATCC 25922.

**Figure 5:**
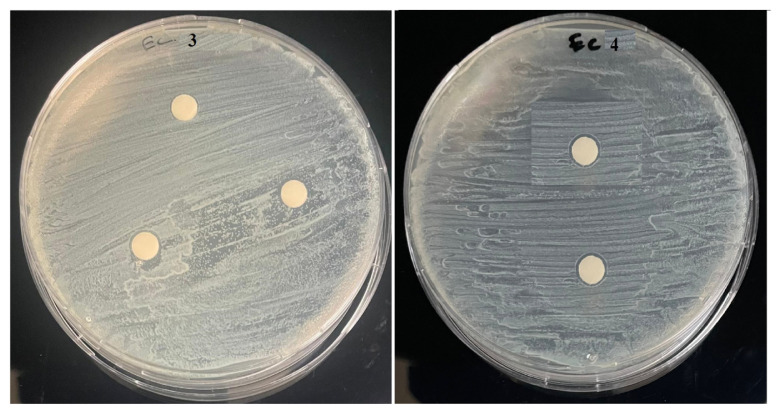
Antibacterial activity of **3–4** against *Escherichia coli* ATCC 25922.

The antibacterial activity of the new Anderson-Evans type POMs (**1–4**) was studied against one gram-positive bacteria (*Staphylcoccus aureus* ATCC 25923) and one gram-negative bacteria *Escherichia coli* ATCC 25922. The results of the antibacterial activities are presented in Table 2 and Figures 2–5. Compound **1–2** have inhibitory action against both microorganisms. But compound **3–4** have no antibacterial activity. This difference in compounds is thought to be due to the counter ion. It has been reported in the literature that POM compounds containing PPh_4_^+^ cation show antimicrobial activity [9]. Compounds **1** and **2** exhibited potent antibacterial activity for *Staphylcoccus aureus* and *Escherichia coli* as the standard drugs Erythromycin 36 mm and 23.5 mm, respectively.

## 4. Conclusion

This paper reports synthesis, characterization, and antibacterial properties of the new four aluminum substituted Anderson-Evans type polyoxometalates. Their structures were identified using elemental analysis, FT-IR, ^1^H NMR, ^31^P NMR and TGA. In antibacterial studies, **1** and **2** have shown high antibacterial activity.

## Supplemental material

Figure S1 FT-IR spectra of **1**.

Figure S2 FT-IR spectra of **2**.

Figure S3 FT-IR spectra of **3**.

Figure S4 FT-IR spectra of **4**.

Figure S5 ^1^H NMR spectra of **1** (DMSO-d_6_, 400 MHz).

Figure S6 ^1^H NMR spectra of **2** (DMSO-d_6_, 400 MHz).

Figure S7 ^1^H NMR spectra of **3** (DMSO-d_6_, 400 MHz).

Figure S8 ^1^H NMR spectra of **4** (DMSO-d_6_, 400 MHz).

Figure S9 ^31^P NMR spectra of **1** (DMSO-d_6_, 400 MHz).

Figure S10 ^31^P NMR spectra of **2** (DMSO-d_6_, 400 MHz).

Figure S11 TGA spectra of **1**.

Figure S12 TGA spectra of **2**.

Figure S13 TGA spectra of **3**.

Figure S14 TGA spectra of **4**.

## Figures and Tables

**Table 1:** Results for the TGA analysis.

Compound	Losses part	Calculated (%)	Experimental (%)	Losses part	Calculated (%)	Experimental (%)
**1**	4 H_2_O	3.46	3.28	3 PPh_4_	48.88	47.85
**2**	4 H_2_O	2.76	2.47	3 PPh_4_	39.00	39.01
**3**	4 H_2_O	5.18	5.56	3 C_7_H_10_N	23.35	23.25
**4**	4 H_2_O	3.76	3.77	3 C_7_H_10_N	23.37	23.28

**Table 2:** Antimicrobial activity of **1–4** against test microorganisms.

Microorganisms (Inhibition zone, mm)^a^	Compounds				Antibiotic	Control
	1	2	3	4	Erythromycin	DMSO
***Staphylococcus aureus***	35	35	CZ	CZ	36	CZ
***Escherichia coli***	23.66	24	CZ	CZ	23.5	CZ

a Inhibition zone diameter in millimeters, CZ: contact zone.

## References

[1] Long D, Tsunashima R, Cronin L (2010). Polyoxometalates: building blocks for functional nanoscale systems. Angewandte Chemie.

[2] Zhang J, Huang Y, Li G, Wei Y (2019). Recent advances in alkoxylation chemistry of polyoxometalates: from synthetic strategies, structural overviews to functional applications. Coordination Chemistry Reviews.

[3] Emirdağ Eanes M, Önen B (2013). Hydrothermal synthesis and characterization of a novel supramolecular hybrid based on Keggin and Cu(I) complex. Inorganic Chemistry Communications.

[4] Emirdağ Eanes M, Önen B, McMillen CD (2015). Hydrothermal synthesis and characterization of one dimensional chain structures of monolacunary Keggin polyoxoanions substituted with copper. Inorganica Chimica Acta.

[5] Wu P, Wang Y, Huang B, Xiao Z (2021). Anderson-type polyoxometalates: from structures to functions. Nanoscale.

[6] Baızıg J, Haj Abdallah A, Haddad A (2018). A new member of Mo36-polyoxoanion’s family: synthesis, crystal structure, and physico-chemical properties of K_10_[Mo_36_O_110_ (OH)_6_ (H_2_O)_12_].38 H_2_O. Turkish Journal of Chemistry.

[7] Blazevic A, Rompel A (2026). The Anderson–Evans polyoxometalate: from inorganic building blocks via hybrid organic–inorganic structures to tomorrows “Bio-POM”. Coordination Chemistry Reviews.

[8] Wang SS, Yang GY (2015). Recent advances in polyoxometalate-catalyzed reactions. Chemical Reviews.

[9] Avcı Özbek H, Kopar E, Demirhan F (2021). Synthesis, structure, and antimicrobial properties of mixed-metal organometallic polyoxometalates [Cp*_2_M_5_VO_17_]^−^ (M=Mo, W). Journal of Coordination Chemistry.

[10] Bijelic A, Rompel A (2015). The use of polyoxometalates in protein crystallography – An attempt to widen a well-known bottleneck. Coordination Chemistry Reviews.

[11] Liu Q, Su T, Zhang H, Liao W, Ren W (2023). Development of TiO_2_ catalyst based on Anderson-polyoxometalates for efficient visible-light-driven photocatalytic oxidative desulfurization. Fuel.

[12] Zhang Y, Chen YZ, Chang ZH, Liu QQ, Wang XL (2022). Metal-directed two new Anderson-type polyoxometalate-based metal–organic complexes with different electrocatalytic sensing performance. Polyhedron.

[13] Ashfaq HF, Ahmad K, Tariq M, Asif HM, Ahmed MM (2022). Synthesis of α-Anderson polyoxometalates-porphyrin polymeric hybrid as an efficient photosensitizer. Polyhedron.

[14] Böke CP, Karaman O, Medetalibeyoglu H, Karamand C, Atar N (2020). A new approach for electrochemical detection of organochlorine compound lindane: development of molecular imprinting polymer with polyoxometalate/carbon nitride nanotubes composite and validation. Microchemical Journal.

[15] Streb C, Kastner K, Tucher J (2019). Polyoxometalates in photocatalysis. Physical Sciences Reviews.

[16] Denghani R, Aber S, Mahdizadeh F (2018). Polyoxometalates and their composites as photocatalysts for organic pollutants degradation in aqueous media-a review. Clean – Soil, Air, Water.

[17] Yola ML, Göde C, Atar N (2017). Molecular imprinting polymer with polyoxometalate/carbon nitride nanotubes for electrochemical recognition of bilirubin. Electrochimica Acta.

[18] Olgun A, Çolak AT, Gübbük IH, Şahin O, Kanar E (2017). A new Keggin-type polyoxometalate catalyst for degradation of aqueous organic contaminants. Journal of Molecular Structure.

[19] Rafiee E, Rahpeyma N (2016). Enhanced delivery of epirubicin by polyoxometalate-based magnetic nanocarriers: controlled drug loading and pH-sensitive drug release. Turkish Journal of Chemistry.

[20] Eren T, Atar N, Yola ML, Karimi-Maleh H, Çolak AT (2015). Facile and green fabrication of silver nanoparticles on a polyoxometalate for Li-ion battery. Ionics.

[21] Li HL, Zhang M, Lian C, Lang ZL, Lv H (2020). Ring-shaped polyoxometalate built by {Mn_4_PW_9_} and PO_4_ units for efficient visible-light-driven hydrogen evolution. Chinese Chemical Society Chemistry.

[22] Zheyu W, Yalin C, Han Y, Sheng H, Yongge W (2020). Application of Anderson type heteropoly acids as catalysts in organic synthesis. Acta Chimica Sinica.

[23] Zang D, Wang H (2022). Polyoxometalate-based nanostructures for electrocatalytic and photocatalytic CO_2_ reduction. Polyoxometalates.

[24] Xia Z, Wang L, Zhang Q, Li F, Xu L (2022). Fast degradation of phenol over porphyrin-polyoxometalate composite photocatalysts under visible light. Polyoxometalates.

[25] Zhang Y, Wang X, Wang Y, Xu N, Wang XL (2022). Anderson-type polyoxometalate-based sandwich complexes bearing a new “V”-like bis-imidazole-bis-amide ligand as electrochemical sensors and catalysts for sulfide oxidation. Polyoxometalates.

[26] He P, Ran L, Huang R, Hu R, Ma R (2022). Old molybdenum blue for new application: {Mo72X30}/PANI/MWCNTs (X = Fe, V) ternary coaxial cable-like fibers for superior electromagnetic wave absorption. Polyoxometalates.

[27] Zhang H, Zhao WL, Li H, Zhuang Q, Sun Z (2022). Latest progress in covalently modified polyoxometalates-based molecular assemblies and advanced materials. Polyoxometalates.

[28] Li J, Zhang D, Chi Y, Hu C (2022). Catalytic application of polyoxovanadates in the selective oxidation of organic molecules. Polyoxometalates.

[29] Wei Y (2022). Polyoxometalates: An interdisciplinary journal focused on all aspects of polyoxometalates. Polyoxometalates.

[30] Zhang Q, Li F, Xu L (2023). Application of polyoxometalates in third-generation solar cells. Polyoxometalates.

[31] Cheng D, Gao Z, Wang W, Li S, Li B (2023). Zwitterion-dissociated polyoxometalate electrolytes for solid-state supercapacitors. Polyoxometalates.

[32] Wu P, Yin P, Zhang J, Hao J, Xiao Z (2011). Single-side organically functionalized Anderson-type polyoxometalates. Chemistry A European Journal.

[33] Zhang J, Huang Y, Zhang J, She S, Hao J (2014). A direct anchoring of Anderson-type polyoxometalates in aqueous media with tripodal ligands especially containing the carboxyl group. Dalton Transactions.

[34] Zhang J, Liu Z, Huang Y, Zhang J, Hao J (2015). Unprecedented χ isomers of single-side triol-functionalized Anderson polyoxometalates and their proton-controlled isomer transformation. Chemical Communications.

[35] Zarnegaryan A (2022). Facile synthesis of polyoxometalate supported on magnetic graphene oxide as a hybrid catalyst for efficient oxidation of aldehydes. Scientific Reports.

[36] Tewari S, Adnan M, Balendra, Kumar V, Jangra G (2019). Photoluminescence properties of two closely related isostructural series based on Anderson-Evans cluster coordinated with lanthanides [Ln(H_2_O)_7_{X(OH)_6_Mo_6_O_18_}]·yH_2_O, X = Al, Cr. Frontiers in Chemistry.

[37] Thabet S, Ayed D, Haddad A (2013). novel organic-inorganic hybrid with Anderson type polyanions as building blocks: (C_6_H_10_N_3_O_2_)_2_Na(H_2_O)_2_[Al(OH)_6_Mo_6_O_18_]·6H_2_O. Materials Research Bulletin.

[38] Zhou Y, Yin J, Zhang L (2009). Counter cation Al^3+^ assisting formation of a 2D water sheet that contains both cyclic water hexamers in boat and chair conformations and cyclic water tetramers in an Anderson-type polyoxometalate [Al(H_2_O)_6_][Al(OH)_6_Mo_6_O_18_]·10H_2_O. Journal of Molecular Structure.

[39] Gavrilova LO, Molchanov VN (2005). Heteropoly complexes Na_0.5_Cs_2-x_[H_0.5-x_M^II^_x_XIIl(OH)_6_Mo_6_O_18_]·7-8H_2_O (M^II^=Fe, Mn; X^III^= Cr,Al). Russian Journal of Coordination Chemistry.

[40] Shivaiah V, Das SK (2005). Polyoxometalate-supported transition metal complexes and their charge complementarity: Synthesis and characterization of [M(OH)_6_Mo_6_O_18_{Cu(Phen)(H_2_O)_2_}_2_][M(OH)_6_Mo_6_O_18_{Cu(Phen)(H_2_O)Cl}_2_]·5H_2_O(M=Al^3+^, Cr^3+^). Inorganic Chemistry.

[41] Shivaiah V, Nagaraju M, Das SK (2003). Formation of a spiral-shaped inorganic-organic hybrid chain, [Cu^II^(2,2’-bipy)(H_2_O)_2_Al(OH)_6_Mo_6_O_18_]_n_^n−^: influence of intra- and interchain supramolecular interactions. Inorganic Chemistry.

[42] Cao R, Liu S, Xie L, Pan Y, Cao J (2008). Influence of different site symmetries of Eu^3+^ centers on the luminescence properties of Anderson-based compounds. Inorganica Chimica Acta.

[43] Dhara S, Dey S, Basu S, Drew MGB, Chattopadhyay P (2007). Separation of ^137m^Ba from ^137^Cs using new ion exchanger Na_2_(H_2_O)_4_(H_3_O)[Al(OH)_6_Mo_6_O_18_]. Radiochimica Acta.

[44] Gumerova NI, Melnik NA, Rozantsev GM, Baumer VN, Radio SV (2015). Sodium heteropolyhexamolybdenumnickelate (II) Na_4_[Ni(OH)_6_Mo_6_O_18_].16H_2_O with an Anderson anion: synthesis and crystal structure. Journal of Structural Chemistry.

[45] Gharah N, Chowdhury K, Mukherjee M, Bhattacharyya R (2008). Synthesis and crystal structure of a mixed valence heteropoly green compound, (PPh_4_)_4_[PMo_12_O_40_], and its use as a catalyst in olefin epoxidation. Transition Metal Chemistry.

