# Supplementary figures and images for: Synthesis and characterization of Anderson-Evans type polyoxometalates, antibacterial properties
Source: Turk J Chem. 2023 Jun 7;47(4):742–8. doi: 10.55730/1300-0527.3575 (PMC10760579; doi:10.55730/1300-0527.3575)

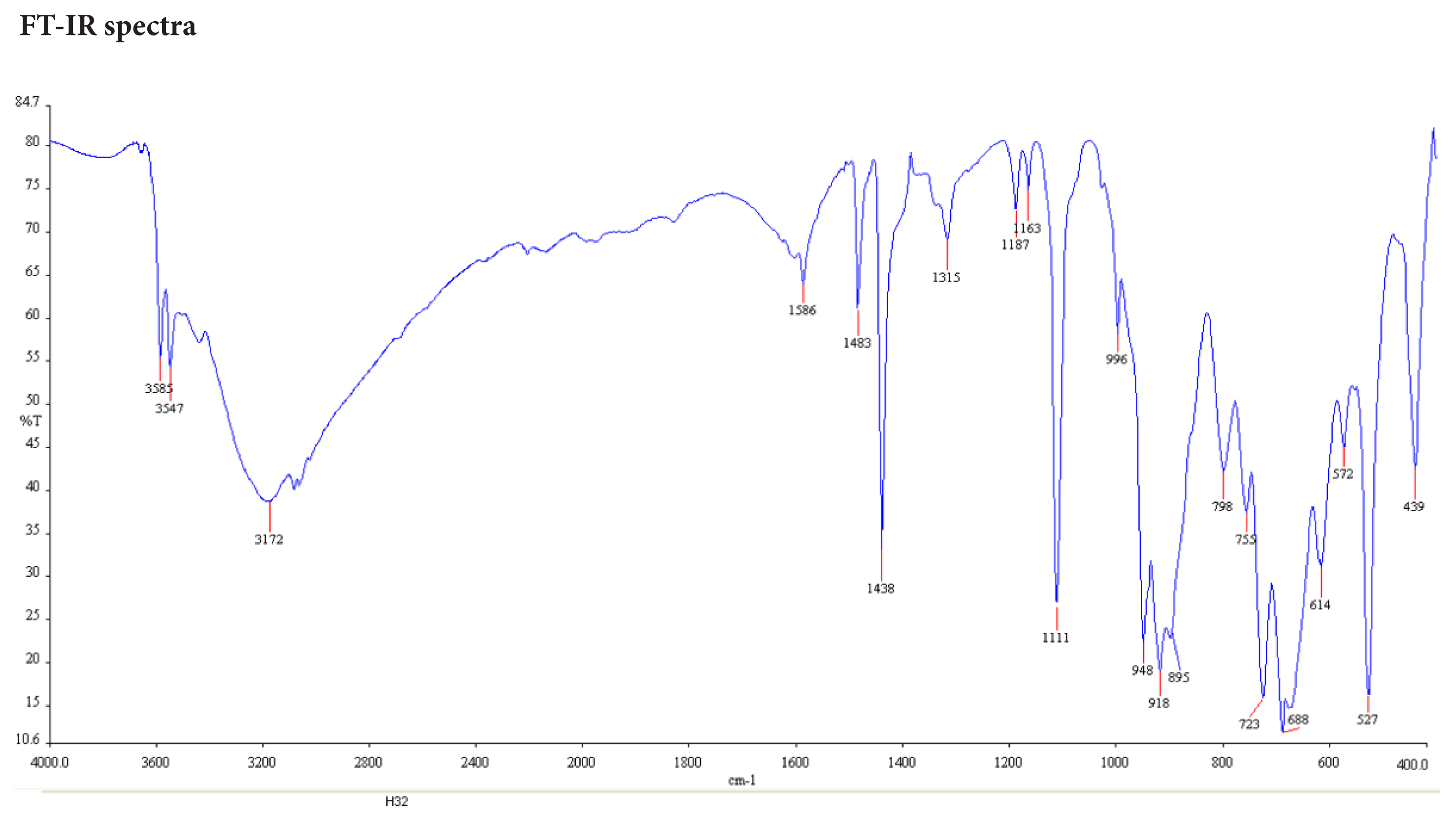

Supplement: Figure S1 — FT-IR spectra of 1. [file turkjchem-47-4-742s1.tif]

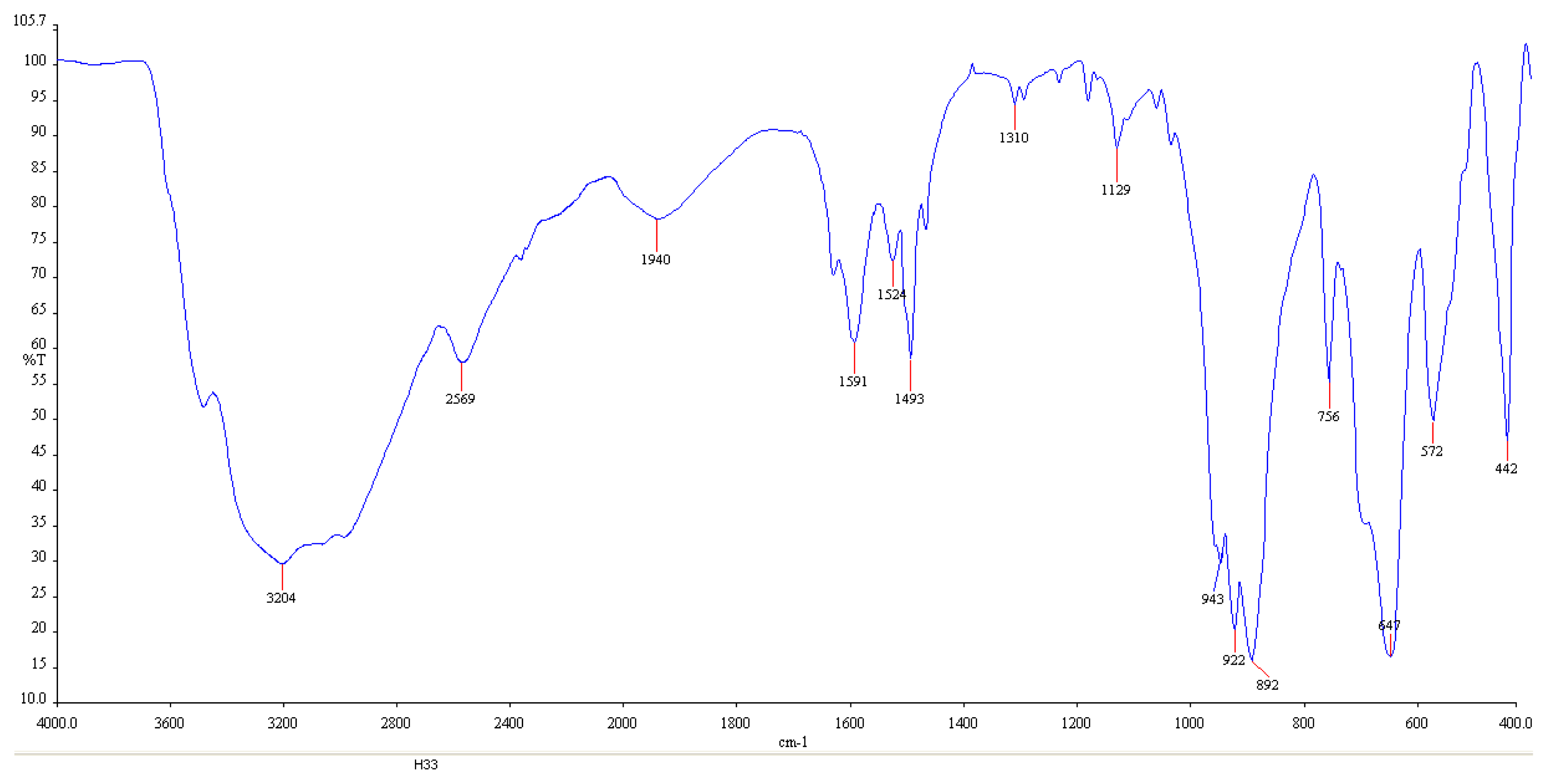

Supplement: Figure S2 — FT-IR spectra of 2. [file turkjchem-47-4-742s2.tif]

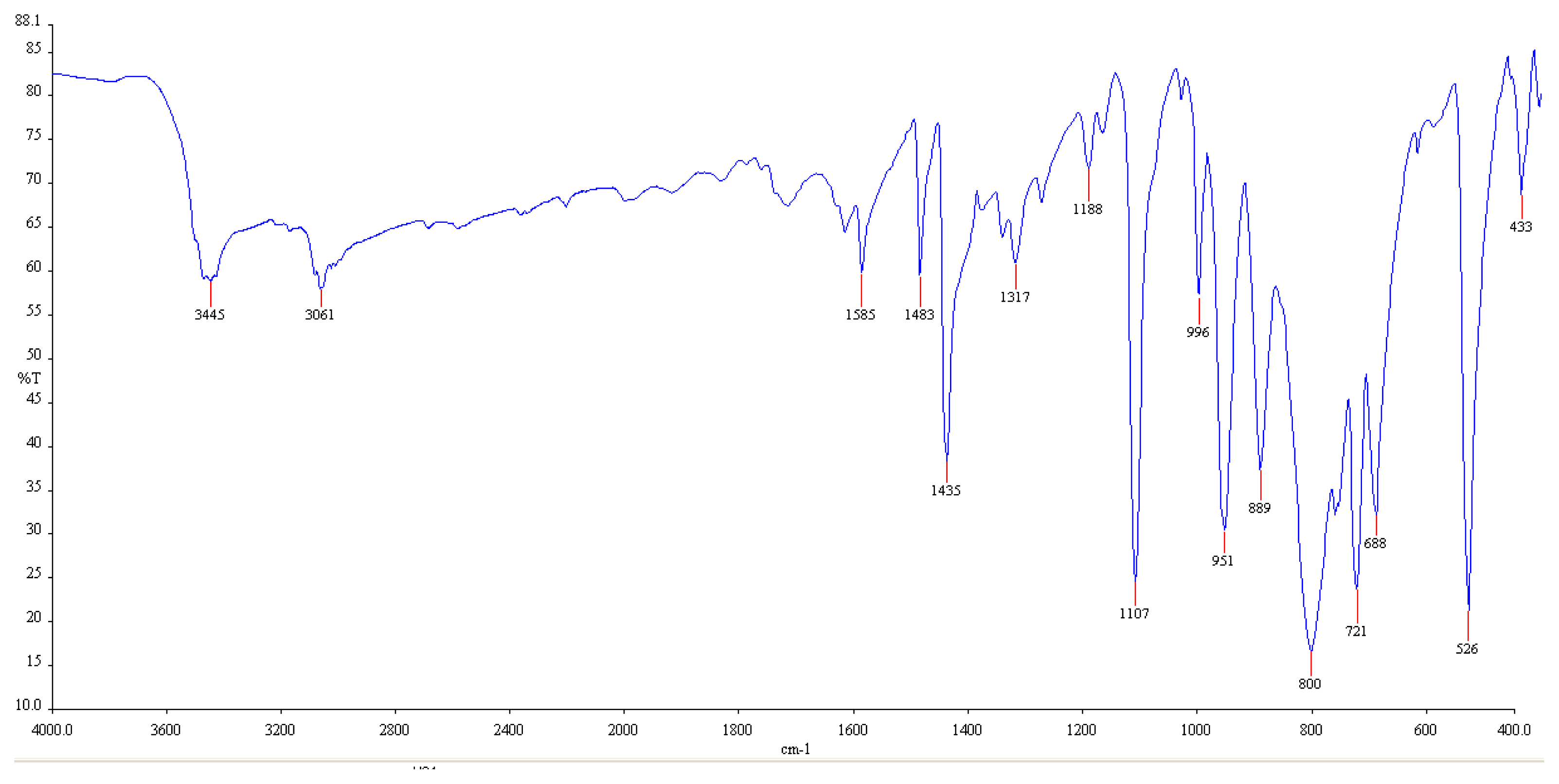

Supplement: Figure S3 — FT-IR spectra of 3. [file turkjchem-47-4-742s3.tif]

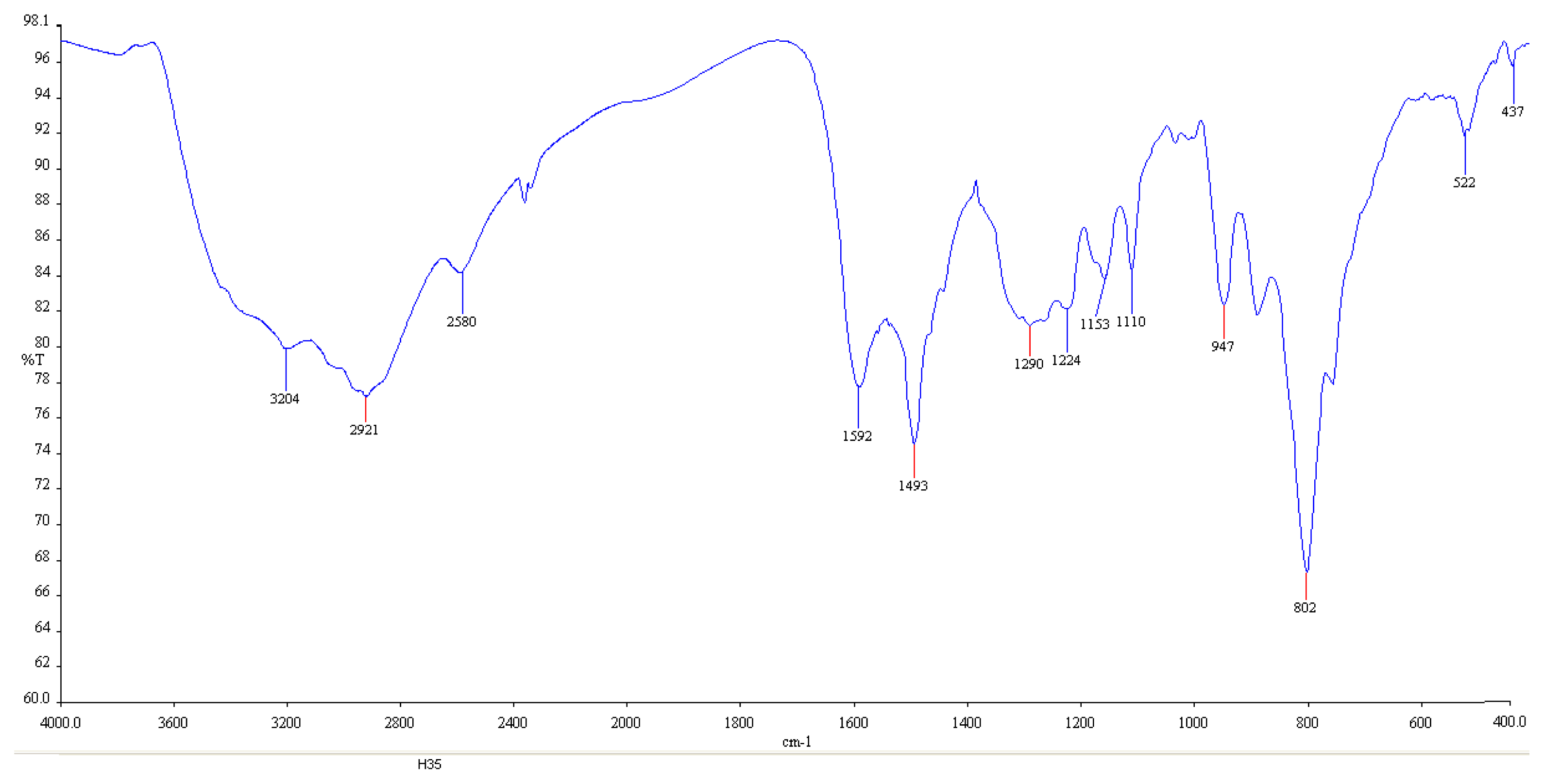

Supplement: Figure S4 — FT-IR spectra of 4. [file turkjchem-47-4-742s4.tif]

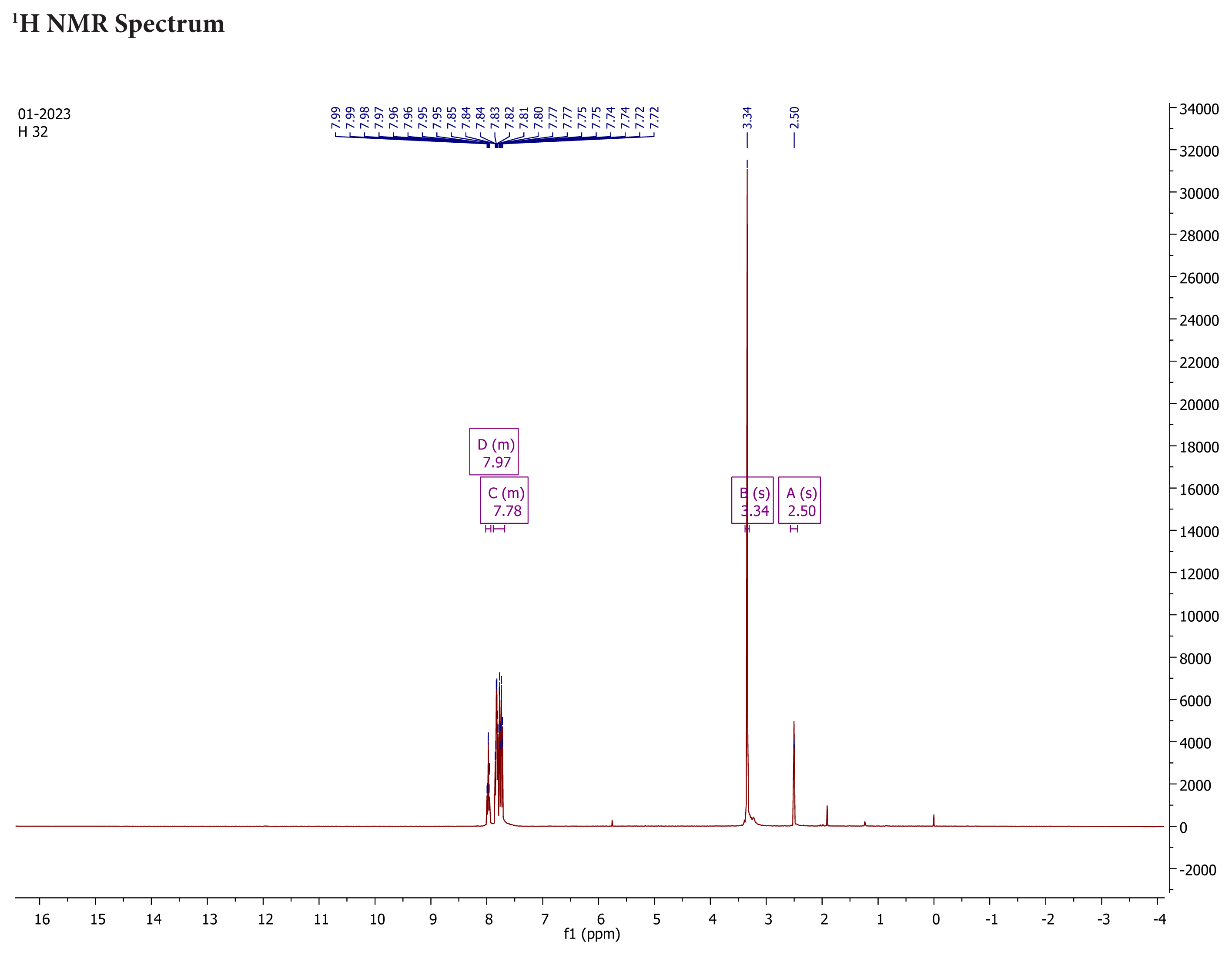

Supplement: Figure S5 — 1H NMR spectra of 1 (DMSO-d6, 400 MHz). [file turkjchem-47-4-742s5.tif]

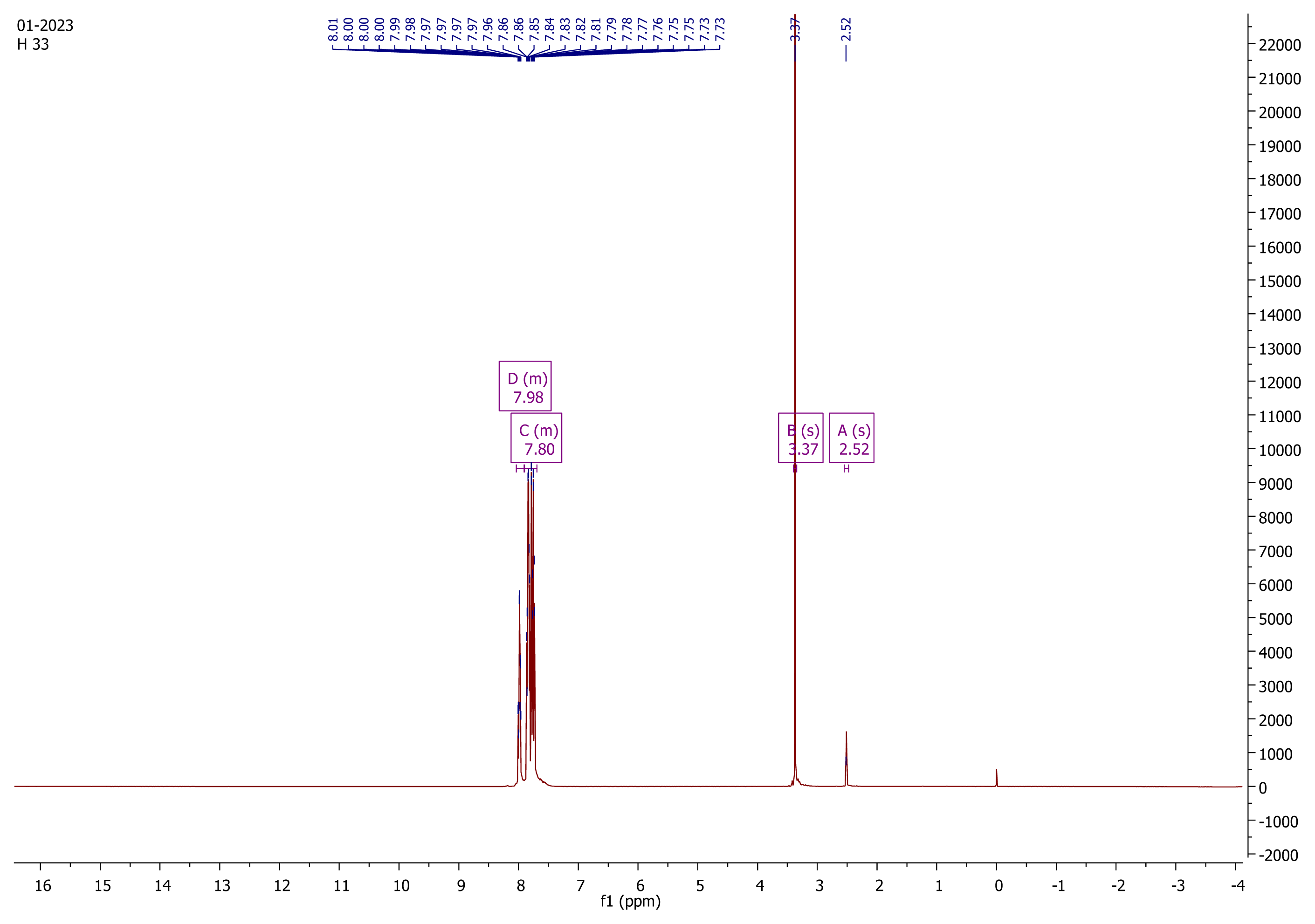

Supplement: Figure S6 — 1H NMR spectra of 2 (DMSO-d6, 400 MHz). [file turkjchem-47-4-742s6.tif]

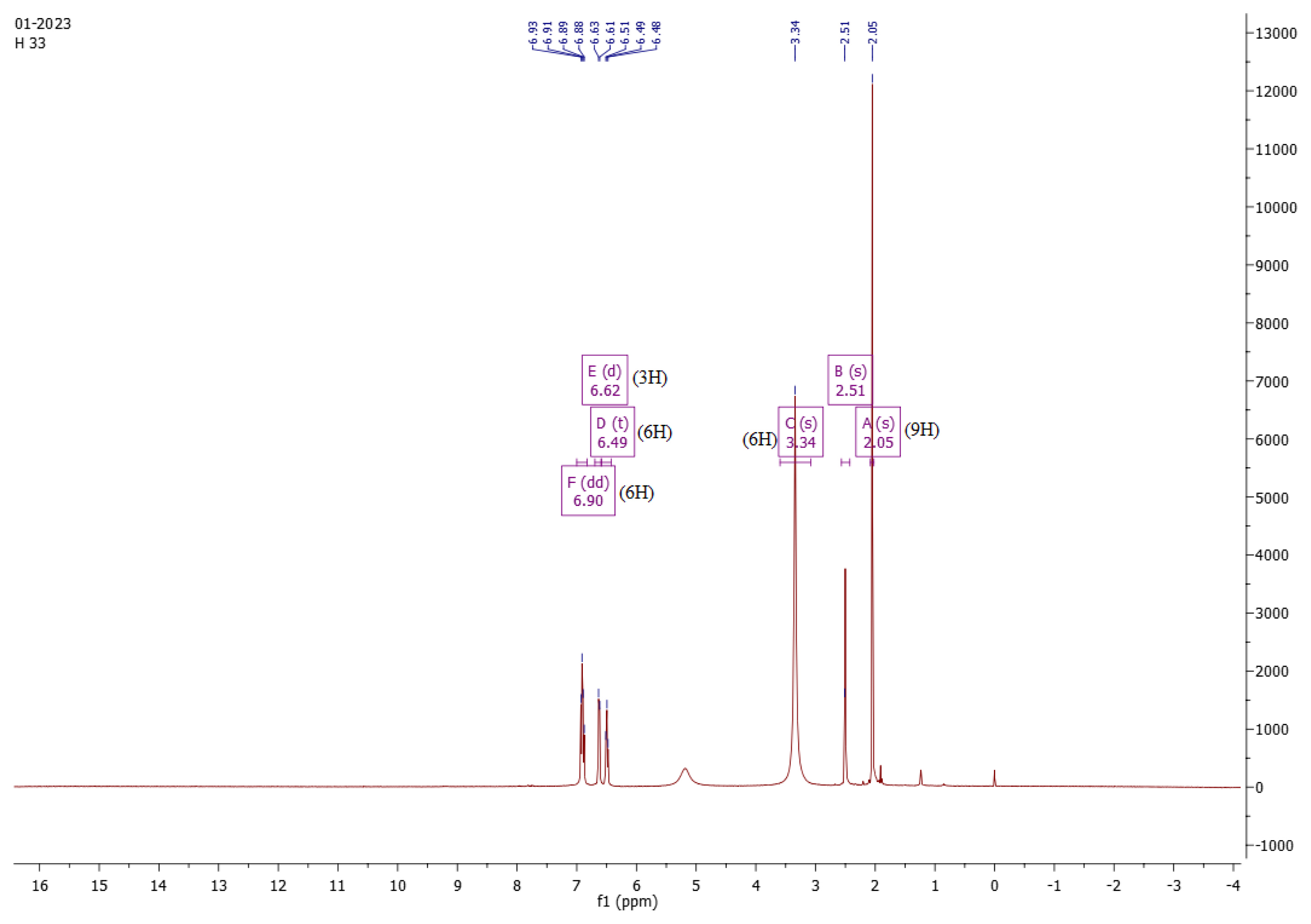

Supplement: Figure S7 — 1H NMR spectra of 3 (DMSO-d6, 400 MHz). [file turkjchem-47-4-742s7.tif]

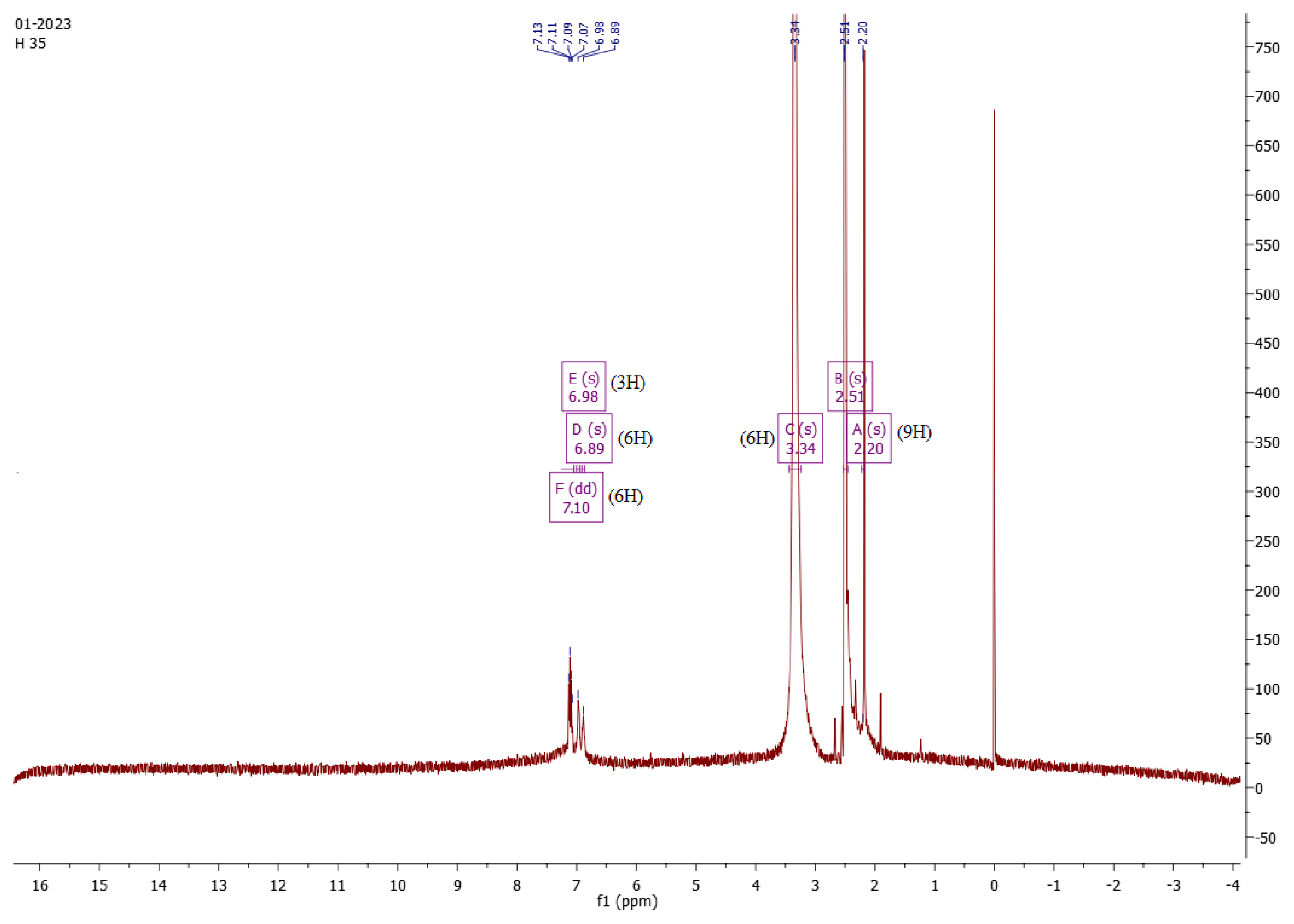

Supplement: Figure S8 — 1H NMR spectra of 4 (DMSO-d6, 400 MHz). [file turkjchem-47-4-742s8.tif]

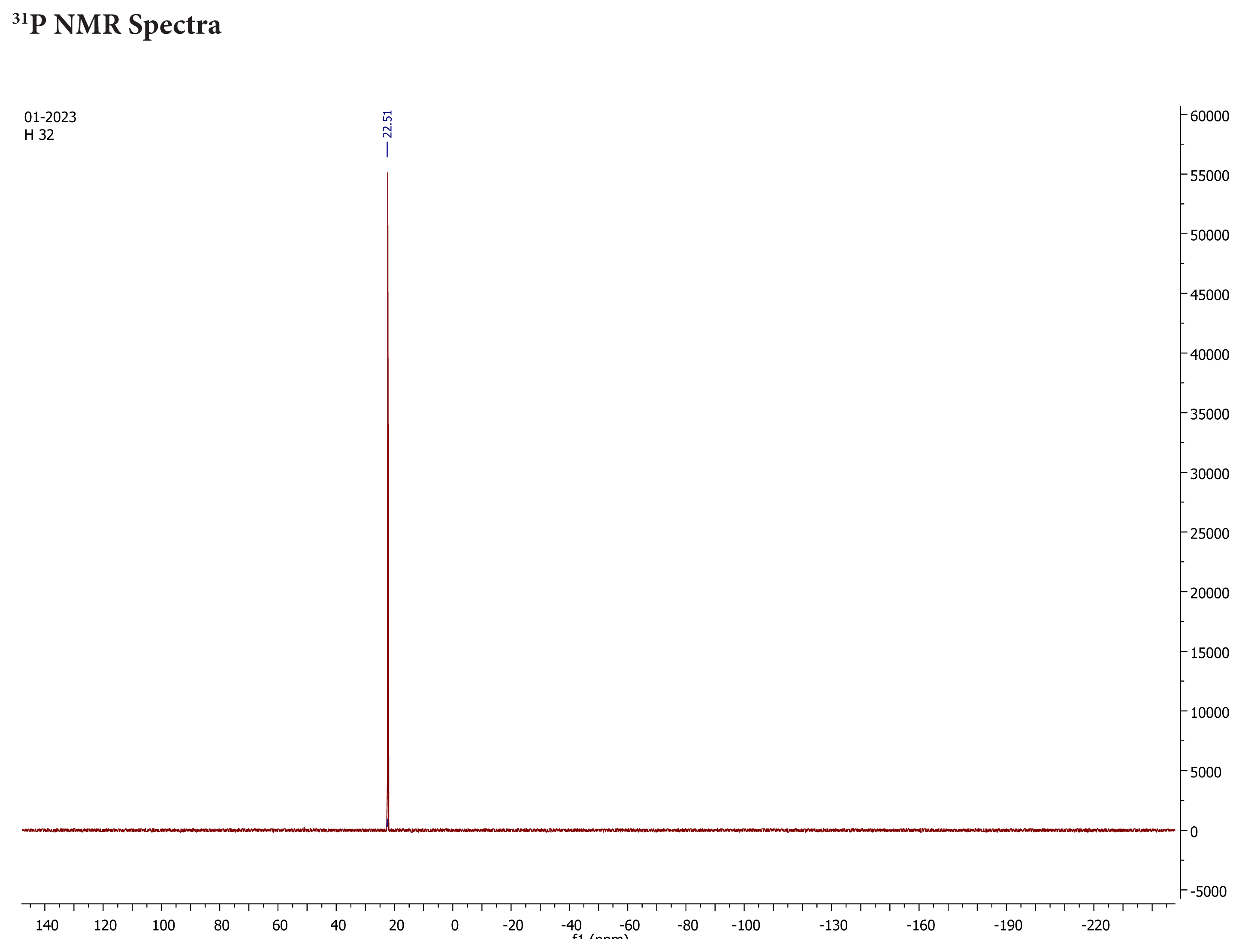

Supplement: Figure S9 — 31P NMR spectra of 1 (DMSO-d6, 400 MHz). [file turkjchem-47-4-742s9.tif]

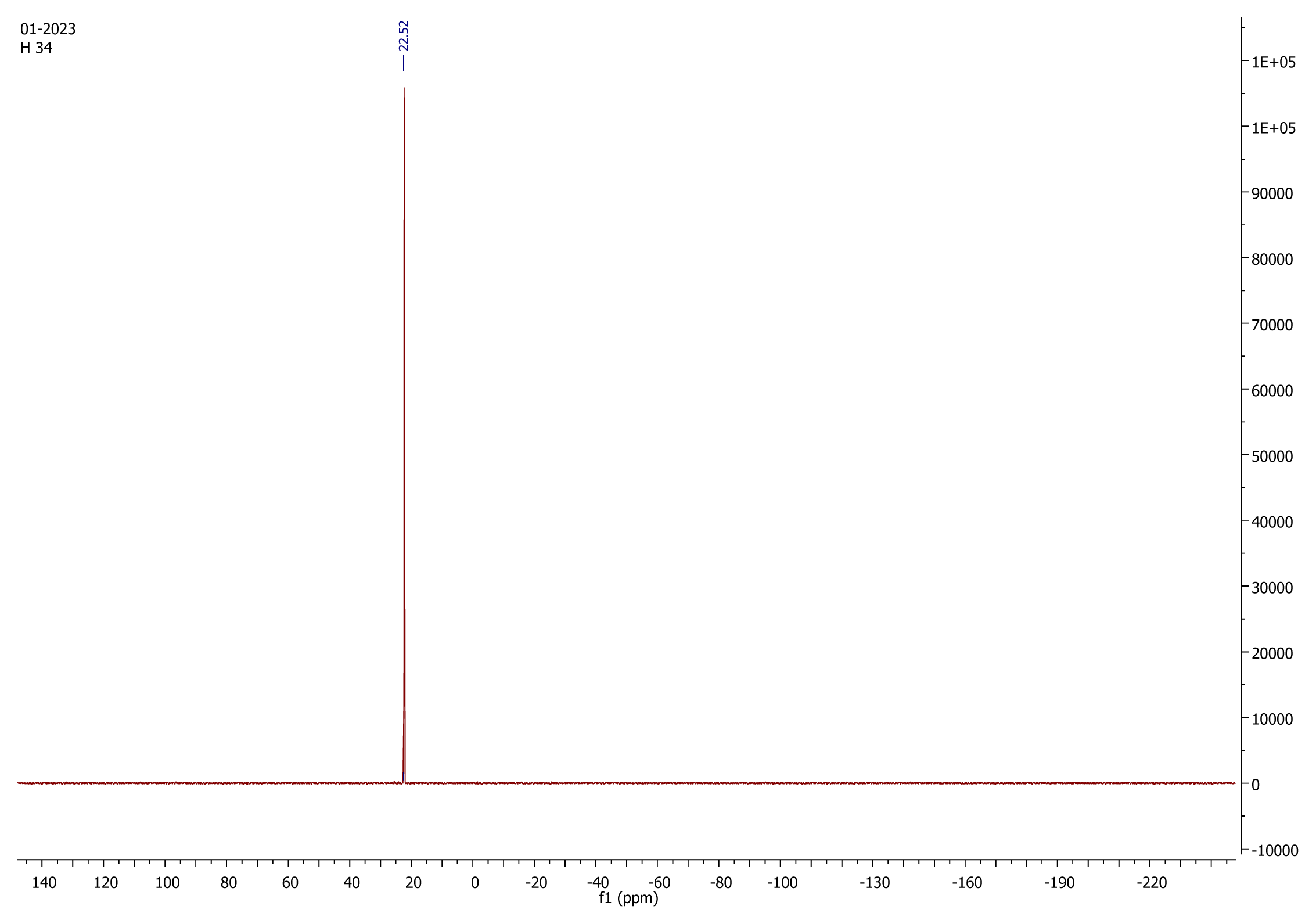

Supplement: Figure S10 — 31P NMR spectra of 2 (DMSO-d6, 400 MHz). [file turkjchem-47-4-742s10.tif]

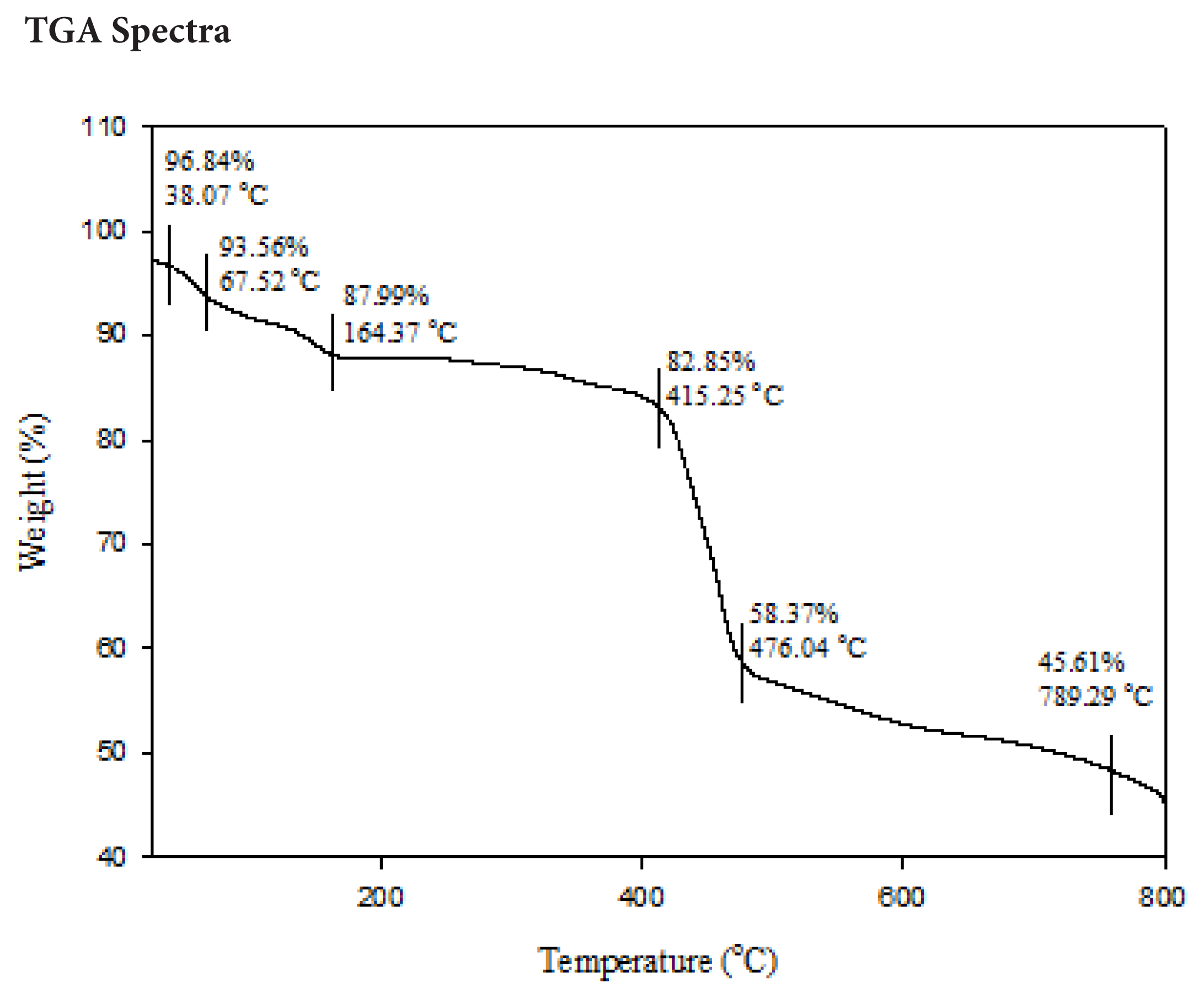

Supplement: Figure S11 — TGA spectra of 1. [file turkjchem-47-4-742s11.tif]

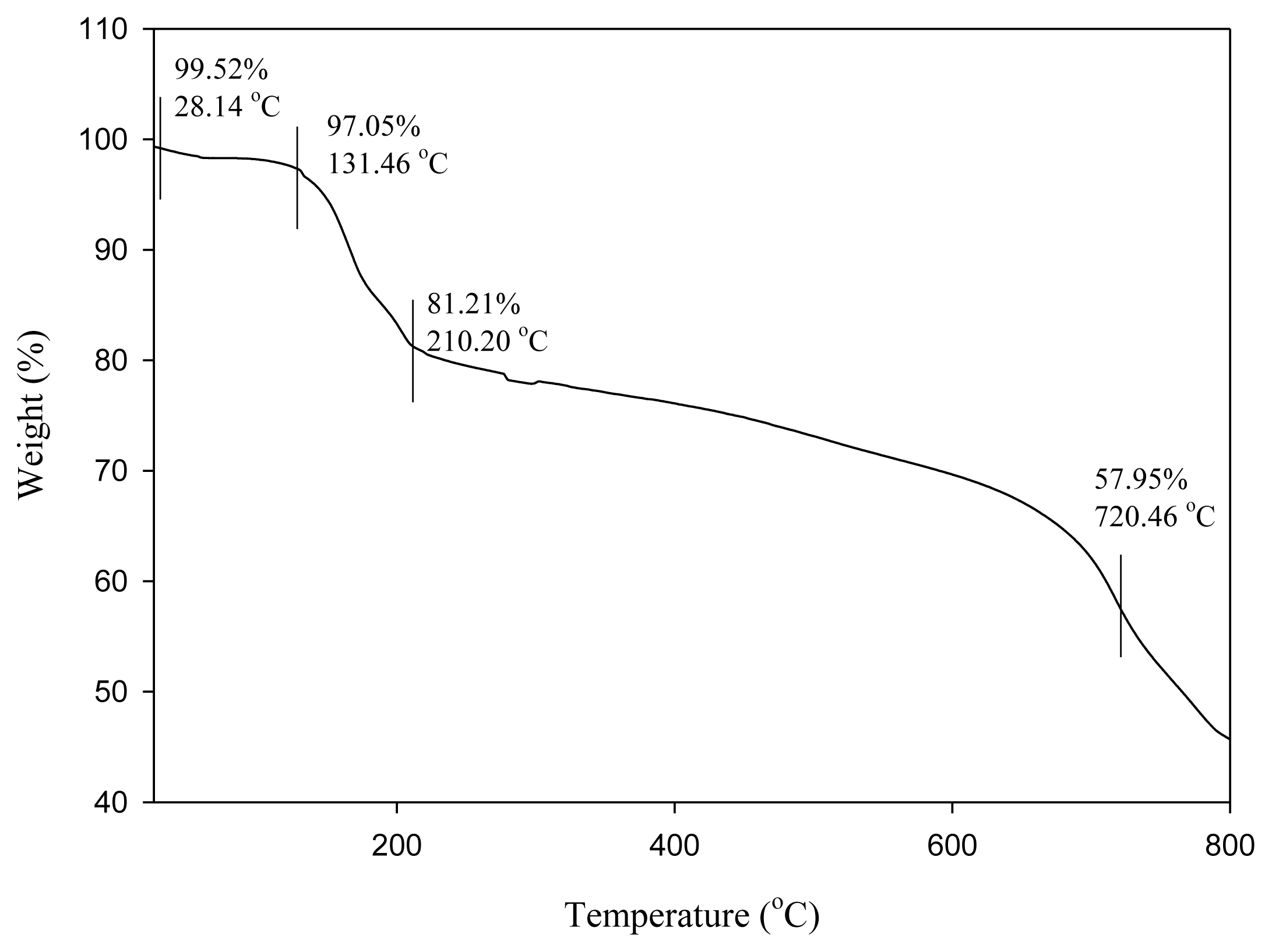

Supplement: Figure S12 — TGA spectra of 2. [file turkjchem-47-4-742s12.tif]

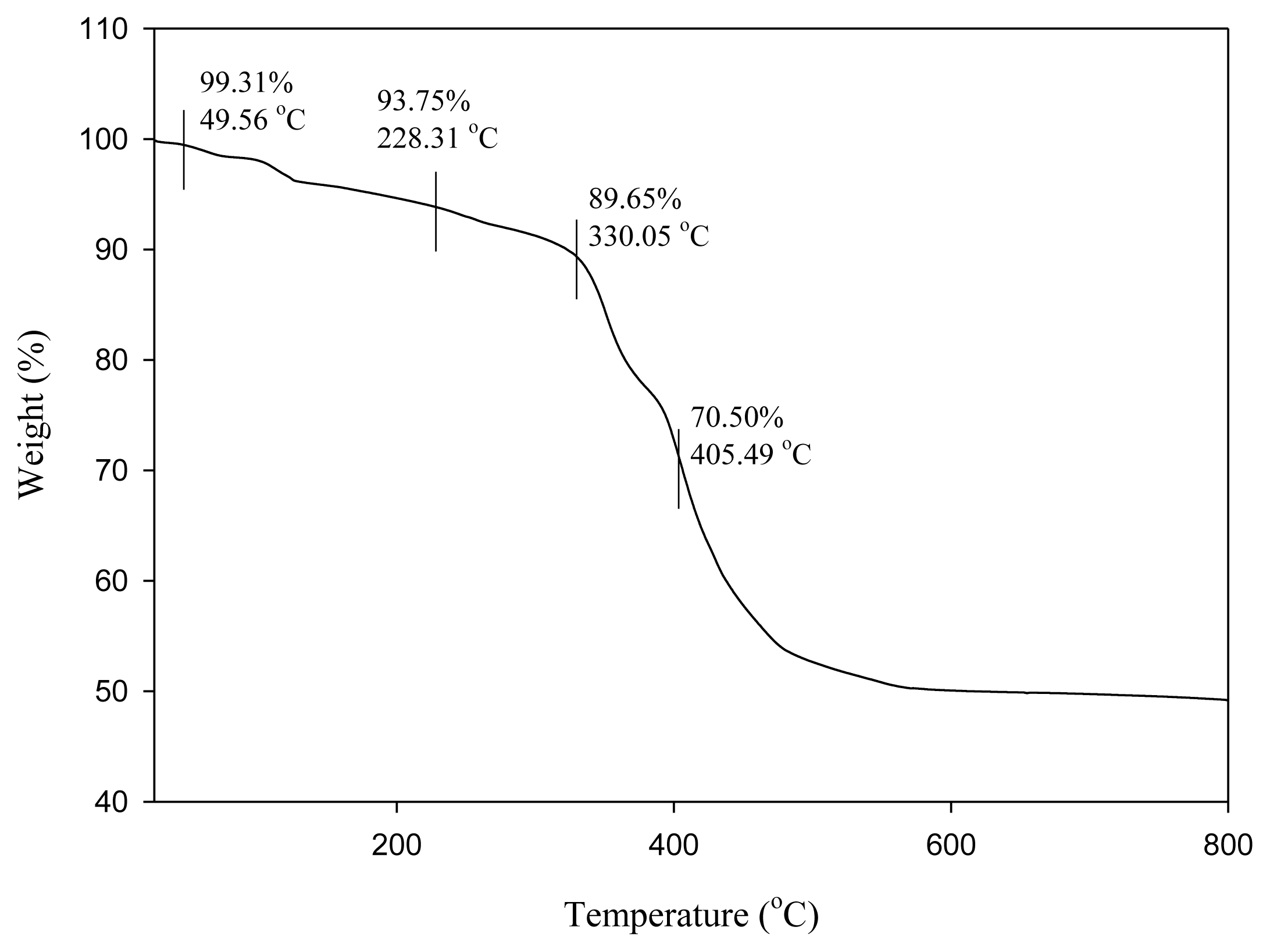

Supplement: Figure S13 — TGA spectra of 3. [file turkjchem-47-4-742s13.tif]

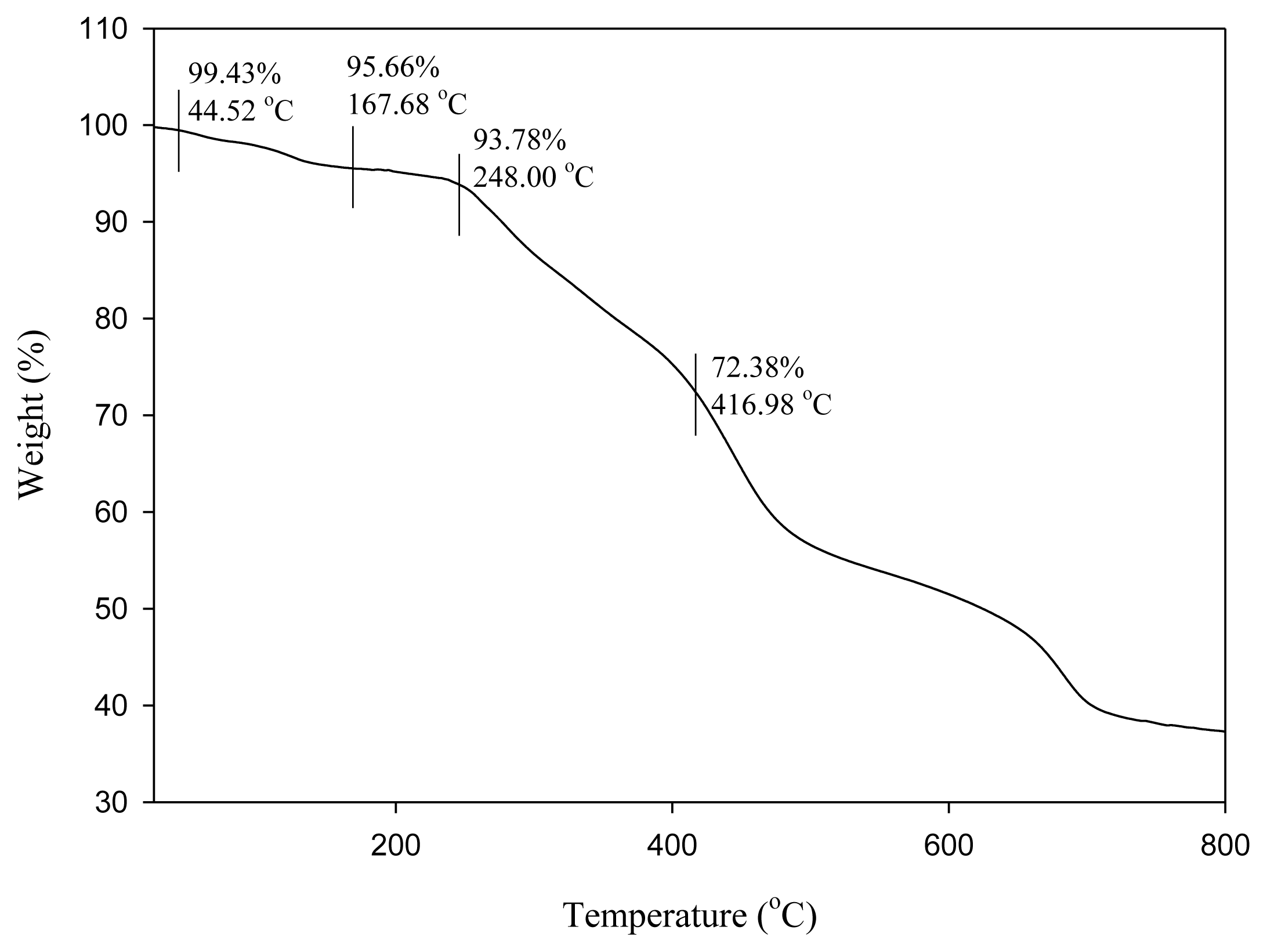

Supplement: Figure S14 — TGA spectra of 4. [file turkjchem-47-4-742s14.tif]
